# Telomerase Activation in Hematological Malignancies

**DOI:** 10.3390/genes7090061

**Published:** 2016-09-07

**Authors:** Joana Ropio, Jean-Philippe Merlio, Paula Soares, Edith Chevret

**Affiliations:** 1 Cutaneous Lymphoma Oncogenesis Team INSERM U1053 Bordeaux Research in Translational Oncology, Bordeaux University, Bordeaux 33076, France; joana.ropio@gmail.com (J.R.); jp.merlio@u-bordeaux.fr (J.-P.M.); 2 Institute of Biomedical Sciences of Abel Salazar, University of Porto, Porto 4050-313, Portugal; 3 Instituto de Investigação e Inovação em Saúde, Universidade do Porto, Porto 4200-135, Portugal; psoares@ipatimup.pt; 4 Institute of Molecular Pathology and Immunology of the University of Porto (Ipatimup)—Cancer Biology, Rua Dr. Roberto Frias, s/n, Porto 4200-465, Portugal; 5 Tumor Bank and Tumor Biology Laboratory, University Hospital Center Bordeaux, Pessac 33604, France; 6 Department of Pathology and Oncology, Medical Faculty of Porto University, Porto 4200-319, Portugal

**Keywords:** telomerase activity, hematological malignancy, amplification, epigenetic, polymorphism, mutation, virus

## Abstract

Telomerase expression and telomere maintenance are critical for cell proliferation and survival, and they play important roles in development and cancer, including hematological malignancies. Transcriptional regulation of the rate-limiting subunit of human telomerase reverse transcriptase gen (*hTERT)* is a complex process, and unveiling the mechanisms behind its reactivation is an important step for the development of diagnostic and therapeutic applications. Here, we review the main mechanisms of telomerase activation and the associated hematologic malignancies.

## 1. Introduction

The ends of all linear genomes are comprised of a unique and genetically stable structure, termed telomere, which preserves genome integrity [1,2]. Due to the “end replication problem”, telomeric sequences shorten after every cell division, triggering the activation of DNA damage pathways that result in senescence and cell death. Telomere erosion and replicative senescence/apoptosis, limits the replicative capacity of cells, which is considered an important tumor-suppressive mechanism [3]. Shortening of telomeres may be counteracted by telomerase, an enzyme specialized in the elongation of telomeric ends [4]. Although the major function of telomerase is telomere elongation, accumulating evidence suggests that telomerase also possess telomere independent functions like enhanced survival, chemo-resistance, invasion and metastasis of malignant cells [5,6,7,8]. 

Telomerase consists of two core components: a catalytic subunit, human telomerase reverse transcriptase (hTERT) with reverse transcriptase activity and an RNA component, human telomerase RNA component (hTR), used as a template for the elongation of telomeres. In vivo telomerase activity requires additional components that associate with hTERT and hTR to form the holoenzyme [9]. Somatic cells do not display detectable telomerase activity, with the exception for germ cells, stem cells and some immune cell types with high proliferative needs. However, in such cells, the telomerase activity is only sufficient to delay, but not to completely prevent telomere shortening [10]. In about 85%–90% of all cancer cells, telomerase is reactivated, allowing the cells to circumvent senescence and divide indefinitely [11]. The remaining cancer cells, preserve the telomere length by a non-telomerase mechanism, known as alternative lengthening of telomeres (ALT), which involves the use of a DNA template [12]. 

It is believed that the limiting factor for telomerase activity is the level of hTERT [13]. Corroborating this idea is the fact that *hTERT* expression is transcriptionally shut-off in somatic cells, but the other telomerase associated components are constitutively expressed in most mammalian cell types; expression of *hTERT* mRNA in somatic cells is sufficient to reconstitute telomerase activity; and the expression levels of *hTERT* show strong correlation with telomerase activity [14,15,16]. 

The expression of *hTERT* is primarily determined by the transcriptional activity of the *hTERT* gene promoter. The *hTERT* promoter does not have typical transcription regulatory elements like TATA and CAAT boxes, but it contains a number of binding sites for multiple important transcription factors, which integrate *hTERT* transcriptional responses with many important pathways that are deregulated in various tumor types [17]. Transcriptional factors and signaling pathways frequently activated in tumor cells, like c-Myc, specific protein 1 (SP1), upstream transcription factor 1 (USF1), signal transducer and activator of transcription 3 (STAT3), phosphoinositide 3-kinase (PI3K), and nuclear factor of activated T-cells (NFAT), can positively stimulate *hTERT* promoter expression. In contrast, Mad, histone deacetylases, E2F1, transforming growth factor-β-activated kinase 1 (TAK1), Wilms' tumor 1 (WT1), p53, mothers against decapentaplegic homolog (Smad3), and Menin signaling negatively regulate *hTERT* promoter expression. [18]. Even if there are a lot of transcription factors involved in telomerase expression regulation, none of them clearly account for the cancer specificity of *hTERT* expression [19]. The *hTERT* gene has a GC rich promoter and may therefore be under epigenetic regulation [20,21]. DNA hypomethylation or histone methylation around the transcription start site of the *hTERT* promoter triggers the recruitment of histone acetyltransferase (HAT) activity, allowing *hTERT* transcription [11]. It is also described in childhood brain tumors that hypermethylation in specific CpG sites upstream of *hTERT* transcription start site, results in telomerase expression [22]. There are also exogenous factors influencing *hTERT* transactivation: several viruses or virus proteins that interact with telomerase are known to be involved in tumorigenesis of infected tissues [23]. Since telomerase activity is a hallmark of the immortal cell phenotype, unveiling the mechanism of telomerase reactivation is an important step for the development of diagnostic and therapeutic applications [24]. This review aims to summarize the mechanisms utilized by hematological malignancies to reactivate telomerase expression.

## 2. Telomeres and Telomerase in Hematologic Malignancies

While *hTERT* expression and telomerase activity are increased in both virus-driven and virus-unrelated lymphoproliferative disorders, telomeres are generally short in virus-unrelated malignancies, and several data suggest that virus-associated tumors and/or pre-neoplastic disorders are characterized by longer telomeres [25,26,27]. This observation may reflect differences in the timing of hTERT activation and, therefore, telomere length stabilization. Indeed, from a theoretical perspective, shorter or longer telomeres could both contribute to oncogenesis [28]. On one hand, long telomeres suggest an early activation of *hTERT* that may contribute to a delay in replicative senescence and prolonged time to acquire genetic alterations critical for the induction of a fully transformed phenotype [23]. On the other hand, telomere shortening ultimately results in genetic instability and activation of *hTERT* may thus occur as a subsequent step, necessary for the immortalization of cells with acquired oncogenic potential. Accordingly, in adult T-cell leukemia/lymphoma (ATLL), telomerase activity appears as a key event in the development and progression of the disease, whereas in acute myeloid leukemia (AML), in chronic myeloid leukemia (CML) and in B-cell diseases, it was demonstrated that telomerase activity is not required for the initiation of disease, but it is required for its maintenance [29,30,31,32]. High telomerase activity is related with progressive disease, worse prognosis, or chemotherapy resistance in the group of hematologic neoplasias [33]. In addition, inhibition of telomerase in leukemia cell lines induces progressive telomere shortening and eventual proliferative arrest or cell death via apoptosis in vitro and in vivo [29,34,35,36]. The observed functional requirement of telomerase in established hematologic malignancies provides a rationale to therapeutically target telomerase in these diseases. 

## 3. Mechanisms of Telomerase Reactivation in Hematologic Malignancies

Although the transcription factors known to bind to *hTERT* promoter may regulate *hTERT* transcription on specific cell type and physiological conditions, none of them are sufficient on their own to promote immortalization of somatic cells [37].

### 3.1. hTERT Amplification 

The *hTERT* gene is frequently amplified in human tumors, including hematological malignancies [38]. In most cases, the amplified region encompassed most or all of the chromosome 5p region. In several cases, chromosomal break points were mapped to regions close to the *hTERT* promoter, suggesting that chromosomal rearrangements could either relieve the promoter from its stringent repressive epigenetic environment or place it in the proximity of enhancers at different chromosomal sites [39,40]. The telomerase reverse transcriptase-cleft lip and palate transmembrane protein 1-like protein (TERT-CLPTM1L) locus including the gene encoding *hTERT* gene is rarely but recurrently targeted by somatic chromosomal translocations to immunoglobulin heavy (IGH) and non-IG loci in B-cell neoplasms, including acute lymphoblastic leukemia, chronic lymphocytic leukemia, mantle cell lymphoma and splenic marginal zone lymphoma. In addition, tumors bearing chromosomal aberrations involving *hTERT* showed higher TERT transcriptional expression and increased telomerase activity [41,42].

Multiple copies of the *hTERT* gene are unlikely to yield a sufficient amount of the *hTERT* transcript, so the marked activation of *hTERT* transcription during tumorigenesis may be a combination of gene amplifications with other genetic or epigenetic mechanisms [37].

### 3.2. Epigenetic Regulation of *hTERT* Gene

Epigenetic modifications imply reversible changes in the genome of cells without any alteration in the DNA sequence. Increasing evidence suggests the epigenetics as an important mechanism involved in cancer initiation, progression, treatment and prognosis. There are three major epigenetic mechanisms that are known to regulate gene transcription in carcinogenesis: modified DNA methylations, histone modifications, and deregulated microRNA (miRNA) expression [43].

The epigenetic plasticity of the *hTERT* gene promoter is a determinant for the control of telomerase activity, and it has been shown that epigenetic modulation may repress *hTERT* transcription in hematological malignancies and may provide an additional level of enzyme regulation [44].

#### 3.2.1. DNA Methylation

There are conflicting studies regarding the correlation between hypermethylation of the *hTERT* promoter, *hTERT* gene expression and telomerase activity [45,46]. 

Regarding hematological malignancies, in a cohort of childhood acute lymphoblastic leukemia (ALL), it was reported that the tumor samples had a methylated promoter, and that the *hTERT* promoter methylation status defines specific ALL subgroups [47]. In addition, in some T cell lymphomas, *hTERT* expression goes along with *hTERT* promoter hypermethylation [48]. It is believed that the promoter hypermethylation interferes with the binding of inhibitors, such as the CTCF transcription factor, which allows the *hTERT* gene to be transcribed [49]. On the contrary, some cells of the lymphoid system seem to escape methylation-dependent mechanism of *hTERT* regulation. Leukemias and lymphomas, including B cell chronic lymphocytic leukemia (CLL), express high levels of telomerase but exhibit low levels of *hTERT* promoter methylation [50]. Moreover, ALL (HL-60) and Burkitt’s lymphoma (Raji) cell lines, were found to have hypomethylated *hTERT* promoters [45,51]. In telomerase-positive B cells, *hTERT* is targeted by paired box 5 (PAX5), a B cell-specific factor, which is sufficient to activate TERT expression [48].

It is believed that the unusual correlations between DNA methylation and expression in cancer cells may, in part, result from the varied methods used to study differing regions of the *hTERT* promoter. Indeed, it was observed that a small region around the transcription start site has little or no methylation, while there is a densely methylated region 600 base pairs upstream the transcription start site [45]. The core promoter demethylation is required for *hTERT* transcription in tumor cells, while the *hTERT* promoter in many normal cells and tissues is either unmethylated or hypomethylated, indicating that *hTERT* silencing does not require extensive CpG methylation at its promoter [46]. In addition, treatment with DNA methyltransferase inhibitors (DNMTIs), including decitabine (DAC) and azacitidine (AZA), is able to cause a reduction in *hTERT* gene expression and telomerase activity [52,53,54]. These two types of drugs are the first molecules that have been approved for the treatment of patients with AML and myelodyplastic syndrome (MDS) [55]. 

#### 3.2.2. Histone Modifications 

Another prevalent epigenetic mechanism that affects *hTERT* transcription is histone modification that includes histone acetylation, methylation, phosphorylation and ubiquitination. Histone deacetylation/methylation, in particular, have been reported to be responsible for the repressive status of the *hTERT* promoter [56]. 

The native chromatin environment is critical for the tight regulation of the *hTERT* gene, as the induction of *hTERT* transcription and telomerase activity in some telomerase-negative cells, was observed upon a treatment with trichostatin A (TSA), a histone deacetylase (HDAC) inhibitor [57,58]. The histone acetylation confers an opened chromatin structure, allowing transcription factors to bind to the DNA [59,60]. HDAC inhibitors are agents that have attracted interest due to their ability to induce not only cell differentiation, but also to promote growth arrest, apoptosis and sensitivity to certain chemotherapeutic reagents. Some HDACs were approved by the United States Food and Drug Administration (U.S. FDA) for the treatment of certain hematological diseases [61]. Modest clinical activity has been reported using HDACs as single-agent therapy, but they appear to be synergistic in vitro and improve response rates in vivo when combined with other agents [62]. Regarding hematological malignancies, the functional impact of HDAC inhibitors on *hTERT* transcription impairment was reported. TSA has an antiproliferative and apoptosis inducing effect on the human leukemic cell line U937, associated with the inhibition of *hTERT* expression and telomerase activity [63]. In leukemia cells it was verified that *hTERT* promoter DNA methylation and histone deacetylation status may contribute to the transcriptional repression of the *hTERT* gene and associated cell differentiation induction, observed during all trans retinoic acid (ATRA) treatment [50,64]. Furthermore, it was reported that the epigenetic modification of the distal domain of the *hTERT* promoter, determines the retinoid capacity to repress telomerase in maturation resistant acute promyelocytic leukemia cells during cellular differentiation [44]. 

Imatinib (IM) is a tyrosine kinase inhibitor selective for the *BCR-ABL* fusion gene, the cytogenetic hallmark of CML [65]. IM administration also inhibits telomerase activity independently of its effect on the BCR-ABL protein, and is mainly caused by hTERT post-translational modifications caused by the downregulation of various members of the phosphatidylinositol-4,5-bisphosphate 3-kinase/protein kinase B (PI3K/AKT) pathway [66,67]. IM resistance is a major problem in therapy and disease relapse of CML. Recent studies have shown that by targeting telomerase expression using a dominant negative form of hTERT, or by the treatment with HDAC inhibitors, the risk of IM resistance may be reduced and the IM induced apoptosis in leukemia cells may be enhanced [68,69].

Histone methylation also plays a role in *hTERT* regulation. SMYD3, a histone methyltransferase (HMTase), is capable to induce *hTERT* transcription and telomerase activity in normal human fibroblasts and cancer cell lines through histone H3 K4 trimethylation [70]. H3-K4 methylation may function as a critical licensing element for transcription factors, such as c-Myc, through which the trans-activation of the *hTERT* gene is initiated [71]. 

#### 3.2.3. MicroRNAs (miRNAs) 

MiRNAs are a family of 19–24 nucleotide non-protein-coding RNA molecules that regulate the stability and translation of target mRNAs [72]. Various studies have revealed that loss- or gain-of- function of specific miRNAs contributes to cellular transformation and tumorigenesis, including hematological diseases [73,74]. The main mechanism that underlies the aberrant miRNA expression is that they are frequently localized in fragile sites prone to translocations and cancer-associated genomic regions (CAGRs) such as minimal regions of loss of heterozygosity (LOH), minimal regions of amplification and common breakpoint regions [75,76]. Many studies propose miRNAs as novel biomarkers and predictors of treatment response of hematological malignancies due to the specific miRNA signatures that allows for discrimination between different subtypes of leukemia and lymphoma with a greater degree of accuracy compared to traditional gene expression analysis [73,77,78]. 

Besides the effort to identify and catalog aberrantly expressed miRNAs in disease, very little is known about the functional consequences of miRNA dysregulation. It has been demonstrated that miRNAs are implicated in the regulation of *hTERT* expression through a regulatory network which is interconnected with other pathways also involved in tumor development. The miRNAs that target the *hTERT* 3′UTR have been identified as tumorigenesis inhibitors, and, consequently, are commonly found downregulated in many types of cancer [79]. The functional impact of miRNAs in B-cell ALL was reported with the restoration of miR-196b expression, which led to significant down-regulation of c-myc and its effector genes, including *hTERT*, suggesting a tumor suppressor function role for miR-196b [80].

Among all the factors regulating telomerase expression and activity, a few polymorphisms and mutations were also identified.

## 4. TERT Polymorphisms 

The presence of single nucleotide variants (SNVs) in the *hTERT* locus, including the promoter and downstream introns, may be an alternative or additional mechanism influencing enzyme’s expression and activity. The genetic variability in the *hTERT* genomic region may affect telomerase function and can modulate telomere length and contribute to the development of cancer as well as the outcome of chemotherapy [81,82]. There are some *TERT* promoter polymorphisms associated with increased risk of developing hematological diseases, and even suggested as prognostic markers of survival [83,84].

Regarding *hTERT* transcription activation, the *TERT* promoter region SNP rs2735940 was associated with risk of childhood ALL. The rs2735940 T allele increased the levels of the *TERT* mRNA compared with the C allele [84]. The role of the mechanism of *hTERT* regulation was also demonstrated via MNS16A polymorphism in patients with Non-Hodgkin Lymphoma (NHL) [85]. The polymorphic element MNS16A, located downstream of exon 16 of the *TERT* gene and upstream of the putative promoter region of an antisense *TERT* transcript, has promoter activity depending on the number of tandem repeats. Carriers of the MNS16A short (S) allele display higher telomerase activity than the long/long (LL) genotype carriers. In fact, longer alleles at MNS16A exhibit stronger promoter activity compared to the shorter alleles, but this leads to increased expression of antisense *TERT* mRNA with a conceivable, or at least partial, silencing of the sense telomerase transcript [86].

## 5. *hTERT* Subunit Mutations

Clinically, loss-of-function mutations in *hTERT* or *hTR* might increase the risk of chemotherapy resistance and predisposal to specific human diseases, like bone marrow failure or dyskeratosis congenita and acquired aplastic anemia, diseases that predispose to MDS and AML [87,88,89]. These clinical observations suggest that telomerase deficiency may contribute to the development of hematopoietic malignancy. Although most cancer cells express telomerase to maintain their proliferative capacity, telomerase deficiency may lead to telomere attrition, hypothesized as a molecular mechanism that promotes genomic instability and predisposal to cancer development including leukemia [90,91]. 

Analysis of MDS/AML cases, secondary to bone marrow failure or dyskeratosis congenita allowed the establishment of an association between *hTERT* mutations and poor prognosis [92,93]. In addition, screening of de novo cases of MDS and AML for telomerase mutations have reported the existence of loss-of-function and non-synonymous mutations in the *hTERT* gene implicated as risk factors for AML [94,95].

## 6. TERT Promoter Mutations

Recently, two hot spot mutations in the *TERT* promoter, (−124 G > A and −146 G > A, C > T on the opposite strand) were reported in several different solid tumors [96,97]. These mutations have strong clinical implications with worse prognosis and poor survival and may represent a novel therapeutic target in solid tumors [96,97,98,99]. However, hematological malignancies are not reported to be subjects for somatic promoter mutations in the *TERT* gene [83,94,97,100].

## 7. Virus-Driven Lymphoid Malignancies 

Viruses involved in lymphomagenesis may directly up-regulate telomerase expression and activity [23,101,102].

### 7.1. HTLV-1-Associated Lymphomas

Infection with the human T-lymphotropic virus type I (HTLV-I) is associated with the development of an aggressive form of T-cell leukemia known as adult T-cell leukemia/lymphoma (ATLL) [103]. High telomerase activity is associated with disease progression, so reactivation of telomerase seems to be a critical event in the development and progression of ATLL [27,104]. HTLV-I virus can directly induce endogenous telomerase up-regulation through the viral protein Tax that plays a central role in the modulation of *hTERT* expression [105]. Indeed, Tax represses *hTERT* promoter in proliferating cells, while it activates it in quiescent cells, allowing the cell cycle progression [106]. Notably, in ATLL cells, Tax expression is very low-to-undetectable, yet these cells retain strong telomerase activity. This suggests that alternative/additional mechanisms, independent of Tax protein, may induce *hTERT* expression and telomerase activity. Additionally, the viral protein HTLV-1 basic leucine zipper (HBZ) expressed in ATLL cells increasese transcriptional activity of JunD, an AP-1 protein, while, HBZ in association with JunD activates the *hTERT* promoter [107]. Furthermore, it has also been shown that interleukin-2 (IL-2) signaling was associated with a PI3K-dependent transcriptional up-regulation of the *hTERT* promoter in HTLV-1 transformed cells. Activation of the PI3K pathway mediates cytoplasmic sequester of the WT1 protein, a strong hTERT promoter suppressor [108]. 

### 7.2. EBV-Associated Lymphoproliferative Disorders

Epstein-Barr virus (EBV) is involved in the pathogenesis of several lymphoproliferative disorders, including Burkitt’s and Hodgkin’s lymphomas, post-transplant lymphoproliferations, and a subset of T/natural killer (NK) cell lymphomas [109]. Among EBV latency gene products, latent membrane protein 1 (LMP-1) is considered the strongest oncoprotein, being essential for immortalization of B cells. A crucial prerequisite for EBV-driven transformation is the induction of latent EBV genes and the down-regulation of lytic EBV gene expression, concomitantly with the induction of *hTERT* expression and activity [110]. *LMP-1* activates *TERT* at the transcriptional level via NF-kB and (Mitogen-Activated Protein Kinase/Extracellular signal-Regulated Kinases) MAPK/ERK1/2 pathways [111].

## 8. Conclusions

The regulation of the *hTERT* gene is a very complex process. The repressed *hTERT* promoter can be activated by multiple mechanisms in different hematological malignancies. In addition to a variety of transcription factors that bind and promote *hTERT* transcription/inhibition, the chromatin environment and nucleosomal conformation appear to be among the major mechanisms that tightly regulate the *hTERT* gene in the majority of hematological malignancies. Through epigenetic modulation, the *hTERT* locus is able to adopt a decondensed state, and then allow the binding of sequence-specific transcription factors that will activate its transcription. Chromosomal translocations of the *hTERT* locus may, in fact, be an important mechanism of telomerase activation, as it allows the escape of the promoter from its native repressive chromatin environment. Amplification of the *hTERT* gene was reported in hematological malignancies, and combined with other genetic or epigenetic mechanisms, results in a marked activation of *hTERT* transcription during tumorigenesis. In virus-driven lymphoid malignancies, the *hTERT* promoter may also be activated directly by viral proteins.

## References

[1] Blackburn E.H. (1991). Structure and function of telomeres. Nature.

[2] Palm W., de Lange T. (2008). How shelterin protects mammalian telomeres. Annu. Rev. Genet..

[3] O’Sullivan R.J., Karlseder J. (2010). Telomeres: Protecting chromosomes against genome instability. Nat. Rev. Mol. Cell Biol..

[4] Blackburn E.H. (2005). Telomeres and telomerase: Their mechanisms of action and the effects of altering their functions. FEBS Lett..

[5] Low K.C., Tergaonkar V. (2013). Telomerase: Central regulator of all of the hallmarks of cancer. Trends Biochem. Sci..

[6] Liu Z., Li Q., Li K., Chen L., Li W., Hou M., Liu T., Yang J., Lindvall C., Björkholm M. (2013). Telomerase reverse transcriptase promotes epithelial–mesenchymal transition and stem cell-like traits in cancer cells. Oncogene.

[7] Nakamura M., Masutomi K., Kyo S., Hashimoto M., Maida Y., Kanaya T., Tanaka M., Hahn W.C., Inoue M. (2005). Efficient inhibition of human telomerase reverse transcriptase expression by RNA interference sensitizes cancer cells to ionizing radiation and chemotherapy. Hum. Gene Ther..

[8] Del Bufalo D., Rizzo A., Trisciuoglio D., Cardinali G., Torrisi M.R., Zangemeister-Wittke U., Zupi G., Biroccio A. (2005). Involvement of hTERT in apoptosis induced by interference with Bcl-2 expression and function. Cell Death Differ..

[9] Blackburn E.H., Collins K. (2011). Telomerase: An RNP enzyme synthesizes DNA. Cold Spring Harb. Perspect. Biol..

[10] Vaziri H., Dragowska W., Allsopp R.C., Thomas T.E., Harley C.B., Lansdorp P.M. (1994). Evidence for a mitotic clock in human hematopoietic stem cells: Loss of telomeric DNA with age. Proc. Natl. Acad. Sci..

[11] Kyo S., Takakura M., Fujiwara T., Inoue M. (2008). Understanding and exploiting *hTERT* promoter regulation for diagnosis and treatment of human cancers. Cancer Sci..

[12] Londoño-Vallejo J.A., Der-Sarkissian H., Cazes L., Bacchetti S., Reddel R.R. (2004). Alternative lengthening of telomeres is characterized by high rates of telomeric exchange. Cancer Res..

[13] Liu Y., Snow B.E., Hande M.P., Yeung D., Erdmann N.J., Wakeham A., Itie A., Siderovski D.P., Lansdorp P.M., Robinson M.O. (2000). The telomerase reverse transcriptase is limiting and necessary for telomerase function in vivo. Curr. Biol..

[14] Meyerson M., Counter C.M., Eaton E.N., Ellisen L.W., Steiner P., Caddle S.D., Ziaugra L., Beijersbergen R.L., Davidoff M.J., Liu Q. (1997). *hEST2*, the putative human telomerase catalytic subunit gene, is up-regulated in tumor cells and during immortalization. Cell.

[15] Bodnar A.G., Ouellette M., Frolkis M., Holt S.E., Chiu C.P., Morin G.B., Harley C.B., Shay J.W., Lichtsteiner S., Wright W.E. (1998). Extension of life-span by introduction of telomerase into normal human cells. Science.

[16] Vaziri H., Benchimol S. (1998). Reconstitution of telomerase activity in normal human cells leads to elongation of telomeres and extended replicative life span. Curr. Biol..

[17] Akincilar S.C., Unal B., Tergaonkar V. (2016). Reactivation of telomerase in cancer. Cell. Mol. Life Sci..

[18] Yaswen P., MacKenzie K.L., Keith W.N., Hentosh P., Rodier F., Zhu J., Firestone G.L., Matheu A., Carnero A., Bilsland A. (2015). Therapeutic targeting of replicative immortality. Semin. Cancer Biol..

[19] Gladych M., Wojtyla A., Rubis B. (2011). Human telomerase expression regulation. Biochem. Cell Biol..

[20] Guilleret I., Benhattar J. (2004). Unusual distribution of DNA methylation within the *hTERT* CpG island in tissues and cell lines. Biochem. Biophys. Res. Commun..

[21] Cong Y.-S., Wen J., Bacchetti S. (1999). The human telomerase catalytic subunit *hTERT*: Organization of the gene and characterization of the promoter. Hum. Mol. Genet..

[22] Castelo-Branco P., Choufani S., Mack S., Gallagher D., Zhang C., Lipman T., Zhukova N., Walker E.J., Martin D., Merino D. (2013). Methylation of the *TERT* promoter and risk stratification of childhood brain tumours: An integrative genomic and molecular study. Lancet Oncol..

[23] Dolcetti R., de Rossi A. (2012). Telomere/telomerase interplay in virus-driven and virus-independent lymphomagenesis: Pathogenic and clinical implications. Med. Res. Rev..

[24] Shay J.W., Wright W.E. (2011). Role of telomeres and telomerase in cancer. Semin. Cancer Biol..

[25] Mochida A., Gotoh E., Senpuku H., Harada S., Kitamura R., Takahashi T., Yanagi K. (2005). Telomere size and telomerase activity in Epstein-Barr virus (EBV)-positive and EBV-negative Burkitt’s lymphoma cell lines. Arch. Virol..

[26] Kamranvar S.A., Gruhne B., Szeles A., Masucci M.G. (2007). Epstein–Barr virus promotes genomic instability in Burkitt's lymphoma. Oncogene.

[27] Kubuki Y., Suzuki M., Sasaki H., Toyama T., Yamashita K., Maeda K., Ido A., Matsuoka H., Okayama A., Nakanishi T. (2005). Telomerase activity and telomere length as prognostic factors of adult T-cell leukemia. Leuk. Lymphoma.

[28] Hackett J.A., Greider C.W. (2002). Balancing instability: Dual roles for telomerase and telomere dysfunction in tumorigenesis. Oncogene.

[29] Röth A., Vercauteren S., Sutherland H.J., Lansdorp P.M. (2003). Telomerase is limiting the growth of acute myeloid leukemia cells. Leukemia.

[30] Vicente-Dueñas C., Barajas-Diego M., Romero-Camarero I., González-Herrero I., Flores T., Sánchez-García I. (2012). Essential role for telomerase in chronic myeloid leukemia induced by BCR-ABL in mice. Oncotarget.

[31] Bruedigam C., Lane S.W. (2016). Telomerase in hematologic malignancies. Curr. Opin. Hematol..

[32] Deville L., Hillion J., Ségal-Bendirdjian E. (2009). Telomerase regulation in hematological cancers: A matter of stemness?. Biochim. Biophys. Acta (BBA)-Mol. Basis Dis..

[33] Jones C.H., Pepper C., Baird D.M. (2012). Telomere dysfunction and its role in haematological cancer. Br. J. Haematol..

[34] Tauchi T., Nakajima A., Sashida G., Shimamoto T., Ohyashiki J.H., Abe K., Yamamoto K., Ohyashiki K. (2002). Inhibition of human telomerase enhances the effect of the tyrosine kinase inhibitor, imatinib, in BCR-ABL-positive leukemia cells. Clin. Cancer Res..

[35] Delhommeau F., Thierry A., Feneux D., Lauret E., Leclercq E., Courtier M.H., Sainteny F., Vainchenker W., Bennaceur-Griscelli A. (2002). Telomere dysfunction and telomerase reactivation in human leukemia cell lines after telomerase inhibition by the expression of a dominant-negative hTERT mutant. Oncogene.

[36] Nakajima A., Tauchi T., Sashida G., Sumi M., Abe K., Yamamoto K., Ohyashiki J.H., Ohyashiki K. (2003). Telomerase inhibition enhances apoptosis in human acute leukemia cells: Possibility of antitelomerase therapy. Leukemia.

[37] Zhu J., Zhao Y., Wang S. (2010). Chromatin and epigenetic regulation of the telomerase reverse transcriptase gene. Protein Cell.

[38] Cao Y., Bryan T.M., Reddel R.R. (2008). Increased copy number of the *TERT* and *TERC* telomerase subunit genes in cancer cells. Cancer Sci..

[39] Zhao Y., Wang S., Popova E.Y., Grigoryev S.A., Zhu J. (2009). Rearrangement of upstream sequences of the *hTERT* gene during cellular immortalization. Genes Chromosom. Cancer.

[40] Zhang A., Zheng C., Lindvall C., Hou M., Ekedahl J., Lewensohn R., Yan Z., Yang X., Henriksson M., Blennow E. (2000). Frequent amplification of the telomerase reverse transcriptase gene in human tumors. Cancer Res..

[41] Nagel I., Szczepanowski M., Martín-Subero J.I., Harder L., Akasaka T., Ammerpohl O., Callet-Bauchu E., Gascoyne R.D., Gesk S., Horsman D. (2010). Deregulation of the telomerase reverse transcriptase (*TERT*) gene by chromosomal translocations in B-cell malignancies. Blood.

[42] Schilling G., Penas E.M., Janjetovic S., Oliveira-Ferrer L., Braig M., Behrmann P., Bokemeyer C., Dierlamm J. (2013). Molecular characterization of chromosomal band 5p15. 33: A recurrent breakpoint region in mantle cell lymphoma involving the TERT–CLPTM1L locus. Leuk. Res..

[43] Dawson M.A., Kouzarides T. (2012). Cancer epigenetics: From mechanism to therapy. Cell.

[44] Azouz A., Wu Y.L., Hillion J., Tarkanyi I., Karniguian A., Aradi J., Lanotte M., Chen G.Q., Chehna M., Ségal-Bendirdjian E. (2010). Epigenetic plasticity of *hTERT* gene promoter determines retinoid capacity to repress telomerase in maturation-resistant acute promyelocytic leukemia cells. Leukemia.

[45] Zinn R.L., Pruitt K., Eguchi S., Baylin S.B., Herman J.G. (2007). *hTERT* is expressed in cancer cell lines despite promoter DNA methylation by preservation of unmethylated DNA and active chromatin around the transcription start site. Cancer Res..

[46] Dessain S.K., Yu H.Y., Reddel R.R., Beijersbergen R.L., Weinberg R.A. (2000). Methylation of the human telomerase gene CpG island. Cancer Res..

[47] Borssén M., Cullman I., Norén-Nyström U., Sundström C., Porwit A., Forestier E., Roos G. (2011). *hTERT* promoter methylation and telomere length in childhood acute lymphoblastic leukemia—Associations with immunophenotype and cytogenetic subgroup. Exp. Hematol..

[48] Bougel S., Renaud S., Braunschweig R., Loukinov D., Morse H.C., Bosman F.T., Lobanenkov V., Benhattar J. (2010). PAX5 activates the transcription of the human telomerase reverse transcriptase gene in B cells. J. Pathol..

[49] Renaud S., Loukinov D., Abdullaev Z., Guilleret I., Bosman F.T., Lobanenkov V., Benhattar J. (2007). Dual role of DNA methylation inside and outside of CTCF-binding regions in the transcriptional regulation of the telomerase *hTERT* gene. Nucleic Acids Res..

[50] Bechter O.E., Eisterer W., Dlaska M., Kühr T., Thaler J. (2002). CpG island methylation of the *hTERT* promoter is associated with lower telomerase activity in B-cell lymphocytic leukemia. Exp. Hematol..

[51] Pettigrew K.A., Armstrong R.N., Colyer H.A., Zhang S.D., Rea I.M., Jones R.E., Baird D.M., Mills K.I. (2012). Differential *TERT* promoter methylation and response to 5-aza-2′-deoxycytidine in acute myeloid leukemia cell lines: *TERT* expression, telomerase activity, telomere length, and cell death. Genes Chromosom. Cancer.

[52] Kumari A., Srinivasan R., Vasishta R.K., Wig J.D. (2009). Positive regulation of human telomerase reverse transcriptase gene expression and telomerase activity by DNA methylation in pancreatic cancer. Ann. Surg. Oncol..

[53] Licht J.D. (2015). DNA Methylation Inhibitors in Cancer Therapy: The Immunity Dimension. Cell.

[54] Guilleret I., Yan P., Grange F., Braunschweig R., Bosman F.T., Benhattar J. (2002). Hypermethylation of the human telomerase catalytic subunit (*hTERT*) gene correlates with telomerase activity. Int. J. Cancer.

[55] Gnyszka A., Jastrzębski Z., Flis S. (2013). DNA methyltransferase inhibitors and their emerging role in epigenetic therapy of cancer. Anticancer Res..

[56] Wang S., Hu C., and Zhu J. (2010). Distinct and temporal roles of nucleosomal remodeling and histone deacetylation in the repression of the *hTERT* gene. Mol. Biol. Cell.

[57] Takakura M., Kyo S., Sowa Y., Wang Z., Yatabe N., Maida Y., Tanaka M., Inoue M. (2001). Telomerase activation by histone deacetylase inhibitor in normal cells. Nucleic Acids Res..

[58] Hou M., Wang X., Popov N., Zhang A., Zhao X., Zhou R., Zetterberg A., Björkholm M., Henriksson M., Gruber A. (2002). The histone deacetylase inhibitor trichostatin A derepresses the telomerase reverse transcriptase (*hTERT*) gene in human cells. Exp. Cell Res..

[59] Krajewski W.A. (2002). Histone acetylation status and DNA sequence modulate ATP-dependent nucleosome repositioning. J. Biol. Chem..

[60] Lai S.R., Phipps S.M., Liu L., Andrews L.G., Tollefsbol T.O. (2004). Epigenetic control of telomerase and modes of telomere maintenance in aging and abnormal systems. Front. Biosci. J. Virtual Libr..

[61] Chun P. (2015). Histone deacetylase inhibitors in hematological malignancies and solid tumors. Arch. Pharm. Res..

[62] Quintas-Cardama A., Santos F., Garcia-Manero G. (2011). Histone deacetylase inhibitors for the treatment of myelodysplastic syndrome and acute myeloid leukemia. Leukemia.

[63] Woo H.J., Lee S.J., Choi B.T., Park Y.M., Choi Y.H. (2007). Induction of apoptosis and inhibition of telomerase activity by trichostatin A, a histone deacetylase inhibitor, in human leukemic U937 cells. Exp. Mol. Pathol..

[64] Pendino F., Sahraoui T., Lanotte M., Segal-Bendirdjian E. (2002). A novel mechanism of retinoic acid resistance in acute promyelocytic leukemia cells through a defective pathway in telomerase regulation. Leukemia.

[65] Capdeville R., Buchdunger E., Zimmermann J., Matter A. (2002). Glivec (STI571, imatinib), a rationally developed, targeted anticancer drug. Nat. Rev. Drug Dis..

[66] Uziel O., Fenig E., Nordenberg J., Beery E., Reshef H., Sandbank J., Birenbaum M., Bakhanashvili M., Yerushalmi R., Luria D. (2005). Imatinib mesylate (Gleevec) downregulates telomerase activity and inhibits proliferation in telomerase-expressing cell lines. Bri. J. Cancer.

[67] Mor-Tzuntz R., Uziel O., Shpilberg O., Lahav J., Raanani P., Bakhanashvili M., Rabizadeh E., Zimra Y., Lahav M., Granot G. (2010). Effect of imatinib on the signal transduction cascade regulating telomerase activity in K562 (BCR-ABL–positive) cells sensitive and resistant to imatinib. Exp. Hematol..

[68] Okabe S., Tauchi T., Nakajima A., Sashida G., Gotoh A., Broxmeyer H.E., Ohyashiki J.H., Ohyashiki K. (2007). Depsipeptide (FK228) preferentially induces apoptosis in BCR/ABL-expressing cell lines and cells from patients with chronic myelogenous leukemia in blast crisis. Stem Cells Dev..

[69] Deville L., Hillion J., Pendino F., Samy M., Nguyen E., Ségal-Bendirdjian E. (2011). hTERT promotes imatinib resistance in chronic myeloid leukemia cells: Therapeutic implications. Mol. Cancer Ther..

[70] Liu C., Fang X., Ge Z., Jalink M., Kyo S., Björkholm M., Gruber A., Sjöberg J., Xu D. (2007). The telomerase reverse transcriptase (*hTERT*) gene is a direct target of the histone methyltransferase SMYD3. Cancer Res..

[71] Guccione E., Martinato F., Finocchiaro G., Luzi L., Tizzoni L., Dall’Olio V., Zardo G., Nervi C., Bernard L., Amati B. (2006). Myc-binding-site recognition in the human genome is determined by chromatin context. Nat. Cell Biol..

[72] Fabian M.R., Sonenberg N., Filipowicz W. (2010). Regulation of mRNA translation and stability by microRNAs. Annu. Rev. Biochem..

[73] Lawrie C.H. (2013). MicroRNAs in hematological malignancies. Blood Rev..

[74] Calin G.A., Croce C.M. (2006). MicroRNA signatures in human cancers. Nat. Rev. Cancer.

[75] Calin G.A., Sevignani C., Dumitru C.D., Hyslop T., Noch E., Yendamuri S., Shimizu M., Rattan S., Bullrich F., Negrini M. (2004). Human microRNA genes are frequently located at fragile sites and genomic regions involved in cancers. PNAS.

[76] Rossi S., Sevignani C., Nnadi S.C., Siracusa L.D., Calin G.A. (2008). Cancer-associated genomic regions (CAGRs) and noncoding RNAs: Bioinformatics and therapeutic implications. Mamm. Genome.

[77] Shah N., Bowles K., Rushworth S.A., MacEwan D. (2015). Understanding the role for miRNA in human leukemia. RNA Dis..

[78] Lu J., Getz G., Miska E.A., Alvarez-Saavedra E., Lamb J., Peck D., Sweet-Cordero A., Ebert B.L., Mak R.H., Ferrando A.A. (2005). MicroRNA expression profiles classify human cancers. Nature.

[79] Hrdličková R., Nehyba J., Bargmann W., Bose H.R. (2014). Multiple tumor suppressor microRNAs regulate telomerase and TCF7, an important transcriptional regulator of the Wnt pathway. PLoS ONE.

[80] Bhatia S., Kaul D., Varma N. (2010). Potential tumor suppressive function of miR-196b in B-cell lineage acute lymphoblastic leukemia. Mol. Cell. Biochem..

[81] Melin B.S., Nordfjäll K., Andersson U., Roos G. (2012). *hTERT* cancer risk genotypes are associated with telomere length. Genet. Epidemiol..

[82] Mocellin S., Verdi D., Pooley K.A., Landi M.T., Egan K.M., Baird D.M., Prescott J., De Vivo I., Nitti D. (2012). Telomerase reverse transcriptase locus polymorphisms and cancer risk: A field synopsis and meta-analysis. J. Nat. Cancer Inst..

[83] Mosrati M.A., Willander K., Falk I.J., Hermanson M., Höglund M., Stockelberg D., Wei Y., Lotfi K., Söderkvist P. (2015). Association between *TERT* promoter polymorphisms and acute myeloid leukemia risk and prognosis. Oncotarget.

[84] Sheng X., Tong N., Tao G., Luo D., Wang M., Fang Y., Li J., Xu M., Zhang Z., Wu D. (2013). *TERT* polymorphisms modify the risk of acute lymphoblastic leukemia in Chinese children. Carcinogenesis.

[85] Wysoczanska B., Wrobel T., Dobrzynska O., Mazur G., Bogunia-Kubik K. (2015). Role of the functional MNS16A VNTR-243 variant of the human telomerase reverse transcriptase gene in progression and response to therapy of patients with non-Hodgkin’s B-cell lymphomas. Int. J. Immunogenet..

[86] Wang L., Soria J.C., Chang Y.S., Lee H.Y., Wei Q., Mao L. (2003). Association of a functional tandem repeats in the downstream of human telomerase gene and lung cancer. Oncogene.

[87] Calado R.T., Young N.S. (2008). Telomere maintenance and human bone marrow failure. Blood.

[88] Townsley D.M., Dumitriu B., Young N.S. (2014). Bone marrow failure and the telomeropathies. Blood.

[89] Vinagre J., Pinto V., Celestino R., Reis M., Pópulo H., Boaventura P., Melo M., Catarino T., Lima J., Lopes J.M. (2014). Telomerase promoter mutations in cancer: An emerging molecular biomarker?. Virchows Arch..

[90] De Lange T. (2005). Telomere-related genome instability in cancer. Cold Spring Harb. Symp. Quant. Biol..

[91] Artandi S.E., Chang S., Lee S.L., Alson S., Gottlieb G.J., Chin L., DePinho R.A. (2000). Telomere dysfunction promotes non-reciprocal translocations and epithelial cancers in mice. Nature.

[92] Aalbers A.M., Calado R.T., Young N.S., Zwaan C.M., Wu C., Kajigaya S., Coenen E.A., Baruchel A., Geleijns K., de Haas V. (2013). Telomere length and telomerase complex mutations in pediatric acute myeloid leukemia. Leukemia.

[93] Kirwan M., Vulliamy T., Marrone A., Walne A.J., Beswick R., Hillmen P., Kelly R., Stewart A., Bowen D., Schonland S.O. (2009). Defining the pathogenic role of telomerase mutations in myelodysplastic syndrome and acute myeloid leukemia. Hum. Mutat..

[94] Yan S., Han B., Wu Y., Zhou D., Zhao Y. (2013). Telomerase gene mutation screening and telomere overhang detection in Chinese patients with acute myeloid leukemia. Leuk. Lymphoma.

[95] Calado R.T., Regal J.A., Hills M., Yewdell W.T., Dalmazzo L.F., Zago M.A., Lansdorp P.M., Hogge D., Chanock S.J., Estey E.H. (2009). Constitutional hypomorphic telomerase mutations in patients with acute myeloid leukemia. Proc. Natl. Acad. Sci. USA.

[96] Huang F.W., Hodis E., Xu M.J., Kryukov G.V., Chin L., Garraway L.A. (2013). Highly recurrent *TERT* promoter mutations in human melanoma. Science.

[97] Vinagre J., Almeida A., Pópulo H., Batista R., Lyra J., Pinto V., Coelho R., Celestino R., Prazeres H., Lima L. (2013). Frequency of *TERT* promoter mutations in human cancers. Nat. Commun..

[98] Melo M., da Rocha A.G., Vinagre J., Batista R., Peixoto J., Tavares C., Celestino R., Almeida A., Salgado C., Eloy C. (2014). *TERT* promoter mutations are a major indicator of poor outcome in differentiated thyroid carcinomas. J. Clin. Endocrinol. Metab..

[99] Batista R., Cruvinel-Carloni A., Vinagre J., Peixoto J., Catarino T.A., Campanella N.C., Menezes W., Becker A.P., de Almeida G.C., Matsushita M.M. (2016). The prognostic impact of *TERT* promoter mutations in glioblastomas is modified by the rs2853669 single nucleotide polymorphism. Int. J. Cancer.

[100] Killela P.J., Reitman Z.J., Jiao Y., Bettegowda C., Agrawal N., Diaz L.A., Friedman A.H., Friedman H., Gallia G.L., Giovanella B.C. (2013). *TERT* promoter mutations occur frequently in gliomas and a subset of tumors derived from cells with low rates of self-renewal. Proc. Nat. Acad. Sci..

[101] Bellon M., Nicot C. (2015). Multiple Pathways Control the Reactivation of Telomerase in HTLV-I-Associated Leukemia. Int. J. Cancer Oncol..

[102] Dolcetti R., Giunco S., Dal Col J., Celeghin A., Mastorci K., De Rossi A. (2014). Epstein-Barr virus and telomerase: From cell immortalization to therapy. Infect. Agents Cancer.

[103] Yoshida M., Seiki M., Yamaguchi K., Takatsuki K. (1984). Monoclonal integration of human T-cell leukemia provirus in all primary tumors of adult T-cell leukemia suggests causative role of human T-cell leukemia virus in the disease. Proc. Nat. Acad. Sci..

[104] Uchida N., Otsuka T., Arima F., Shigematsu H., Fukuyama T., Maeda M., Sugio Y., Itoh Y., Niho Y. (1999). Correlation of telomerase activity with development and progression of adult T-cell leukemia. Leuk. Res..

[105] Sinha-Datta U., Horikawa I., Michishita E., Datta A., Sigler-Nicot J.C., Brown M., Kazanji M., Barrett J.C., Nicot C. (2004). Transcriptional activation of *hTERT* through the NF-κB pathway in HTLV-I–transformed cells. Blood.

[106] Hara T., Matsumura-Arioka Y., Ohtani K., Nakamura M. (2008). Role of human T-cell leukemia virus type I Tax in expression of the human telomerase reverse transcriptase (*hTERT*) gene in human T-cells. Cancer Sci..

[107] Kuhlmann A.S., Villaudy J., Gazzolo L., Castellazzi M., Mesnard J.M., Dodon M.D. (2007). HTLV-1 HBZ cooperates with JunD to enhance transcription of the human telomerase reverse transcriptase gene (hTERT). Retrovirology.

[108] Bellon M., Nicot C. (2008). Central role of PI3K in transcriptional activation of *hTERT* in HTLV-I–infected cells. Blood.

[109] Dolcetti R., Masucci M.G. (2003). Epstein-Barr virus: Induction and control of cell transformation. J. Cell. Physiol..

[110] Terrin L., Dolcetti R., Corradini I., Indraccolo S., Col J.D., Bertorelle R., Bonaldi L., Esposito G., De Rossi A. (2007). hTERT inhibits the Epstein-Barr virus lytic cycle and promotes the proliferation of primary B lymphocytes: Implications for EBV-driven lymphomagenesis. Int. J. Cancer.

[111] Terrin L., Dal Col J., Rampazzo E., Zancai P., Pedrotti M., Ammirabile G., Bergamin S., Rizzo S., Dolcetti R., De Rossi A. (2008). Latent membrane protein 1 of Epstein-Barr virus activates the *hTERT* promoter and enhances telomerase activity in B lymphocytes. J. Virol..

